# Cerebrovascular ageing: how zebrafish can contribute to solving the puzzle

**DOI:** 10.3389/fphys.2025.1548242

**Published:** 2025-02-10

**Authors:** Guy Malkinson, Catarina M. Henriques

**Affiliations:** ^1^ Université de Lorraine, Inserm, DCAC, Nancy, France; ^2^ Center for Interdisciplinary Research in Biology (CIRB), College de France, CNRS, INSERM, Université PSL, Paris, France; ^3^ Department of Oncology and Metabolism, Healthy Lifespan Institute and MRC-Arthritis Research UK Centre for Integrated Research into Musculoskeletal Ageing, University of Sheffield, Sheffield, United Kingdom; ^4^ Bateson Centre, University of Sheffield, Sheffield, United Kingdom

**Keywords:** zebrafish, brain, telomere, telomerase, cerebrovascular, ageing

## Abstract

The mean life expectancy continues to increase world-wide. However, this extended lifespan trend is not accompanied by health span, or years of healthy life. Understanding the fundamental mechanisms responsible for the switch from health to morbidity with ageing are key to identifying potential therapeutic targets to decrease age-associated morbidity and increase years spent in good health. The leading cause of morbidity in Europe are diseases of the circulatory system and diseases of the nervous system and cognitive disorders are among the top-ten. Cerebrovascular ageing is therefore of particular importance as it links circulatory disease to brain functions, cognition, and behavior. Despite major progress in brain research and related technologies, little is known on how the cerebrovascular network changes its properties as ageing proceeds. Importantly, we do not understand why this is different in different individuals in what concerns rate of dysfunction and its downstream impact on brain function. Here we explore how the zebrafish has evolved as an attractive complementary ageing model and how it could provide key insights to understanding the mechanisms underlying cerebrovascular ageing and downstream consequences.

## 1 Introduction

The mean life expectancy has and continues to increase world-wide. However, this extended lifespan trend is not accompanied by health span or years of healthy life. Data from Eurostat reveals that whereas the average life-expectancy at birth was 83.3 and 77.9 years for women and men, respectively, only an average of ∼63 of those years is spent in health. This means that, with increased numbers of elderly and decreased birth rates, there is an increasing burden of morbidity and healthcare costs in society which are predicted to become unsustainable within the next 50 years, according to the European Commission’s Ageing Reports (Commission, 2015; Commission, 2018). Understanding the fundamental mechanisms behind the switch from health to morbidity with ageing are key to identifying potential therapeutic targets to decrease age-associated morbidity and increase years spent in good health.

A fundamental feature of ageing is its temporal dimension, as it is a process that unfolds over time. Depending on the lifespan of the organism, this process can take weeks, months or years, and current ageing models depict it as a series of gradual changes, rather than rapid abrupt variations, whereby ageing reflects the number of changes that accumulate up to a given time point (Meyer and Schumacher, 2024). While there is an on-going debate on how to describe biological ageing with respect to either calendar or chronological ageing (Ikram, 2024), it is accepted that ageing *per se* entails functional and structural changes that affect the physiology of our tissues, organs and body, and much attention is devoted, in fundamental research and medicine, to understanding these changes with the aim of promoting healthy ageing and treating ageing-related pathologies. Different people age differently, at different rates, and are impacted differently by the process. Moreover, evidence suggests a more heterogenous nature of ageing than previously thought, where different organs in the same individual may exhibit different ageing rates (Oh et al., 2023). Our ability to describe the ageing process by observing any changes in the molecular, structural and functional properties, as they occur, would contribute greatly to our understanding of this process.

The leading cause of morbidity in Europe are diseases of the circulatory system and diseases of the nervous system and cognitive disorders are among the top ten. Cerebrovascular ageing is therefore of particular importance as it links circulatory disease to brain functions, cognition, and behavior. The anatomical and physiological changes that are hallmarks of ageing of the cardiovascular network are a risk factor for cardiovascular (CV) diseases (CVD), with a huge impact on health (Laina et al., 2018). Much of what we know about CV ageing comes from epidemiological studies, showing that ageing is a major non-reversible risk factor for CVD. A major hallmark of vascular ageing is the gradual change of vascular structure and function, resulting in increased arterial stiffening, affecting arterial hemodynamics. Molecular mechanisms that may serve as potential targets to delay this phenomenon include telomere shortening and endothelial ubiquitin proteasome system (Laina et al., 2018). CVD is the leading cause of mortality and morbidity in western societies (Townsend et al., 2015), thus the question of how the cardiovascular network ages is of high relevance for aged people. Of particular concern to CV ageing is understanding the changes that happen in the cerebrovascular network, since alterations in this network may result in substantial impact on neural functions and behavior (Zimmerman et al., 2021). Any reduction in the efficacy of the cerebrovascular system will influence hemodynamics and tissue irrigation, with a significant consequent impact on neural functions, cognitive functions and behavior. Vascular calcification, extracellular matrix alterations and synaptic protein shedding were found to be linked to early cognitive decline (Oh et al., 2023). At the morphological level, ageing of the brain itself is associated with loss of brain volume, thinning of the cortex, degradation of white matter, loss of gyrification and enlarged ventricles. At the histological level, this may be accompanied by shrinking and degeneration of neurons, dendrites and synapses, demyelination, white matter lesions, glial cell activation and small vessel disease (Blinkouskaya et al., 2021). Cerebrovascular ageing may also entail loss of arterial elasticity (arteriosclerosis) and plaque buildup (atherosclerosis), impacting tissue perfusion and hemodynamics and leading to inflammation and consequent ischemia. Some vessels increase their tortuosity, resulting in perturbed hemodynamics, and weakening of the endothelial wall of small vessels can in turn lead to aneurysms and hemorrhages, while capillary density itself may decrease. Importantly, the properties of the brain-unique barrier, the blood-brain-barrier (BBB), may be impacted during ageing. The BBB can become leakier, leading to impaired delivery of nutrients and energy to brain tissue, or impaired clearance of cellular waste, and also via altered and dysfunctional neural-vascular-glial signaling, leading to perturbed cerebrovascular reactivity (Zimmerman et al., 2021).

While ageing has vascular correlates that are apparent at the anatomical and mechanical levels, how exactly these different manifestations appear over time is mostly unknown. Some of the open fundamental questions that need to be addressed are whether there is a typical temporal order to the observed changes, if the time of the onset of ageing is similar across different tissues and organs, what are the molecular and cellular signals that underlie ageing, what are their sources and what triggers their release, (activation or deactivation?) Also, how are neural degradation and vascular stiffening linked in time and space, and whether there are reciprocal signals that serve as feedback in the degradation of these systems? Contemporary research into ageing is hampered to a large extent by the difficulty to study this long process at different biological levels, from the molecular level all the way up to physiology and behaviour. This is furthermore complicated in the case of the brain, which is a complex organ that is difficult to access and to repeatedly measure. The field would benefit from a model that enables the observation of the gradual molecular, structural and physiological changes over- time to help build a more holistic picture of ageing in the brain.

## 2 Zebrafish: from a developmental to adult and disease model

Due to the optical transparency of the zebrafish embryo, and the relatively quick and robust development in its first days of life, zebrafish is mostly used as a model for embryonic development. Seventy percent of human genes have at least one zebrafish orthologue, and vice versa (Howe et al., 2013), highlighting the genetic pertinence of this model. Over the years, zebrafish mutant lines and genetically-stable fluorescent reporter lines have contributed greatly to the understanding of early embryonic development of vertebrates, and specifically of various tissues and organs, including the cardiovascular, nervous and immune systems (Martins et al., 2019), to name a few. Zebrafish is a relevant and useful model for studying human diseases, including osteoporosis, atrial fibrillation, leukemia and autism spectrum disorders (Adhish and Manjubala, 2023). Over the last few years, efforts are devoted to using more advanced developmental stages, namely, juvenile and adult fish. While these efforts are confronted with technical challenges, they are also fruitful, and zebrafish adult fish are successfully being used as disease models in several cases. The ability to induce cell or tissue specific genetic modifications in adult-stage fish, for example with recently developed state-of-the-art UFLIP technologies (Liu et al., 2022), enables to model gene-environment interactions, and to study with greater precision pathologies such as cancer, cardiovascular diseases, infectious conditions, toxicology and social behaviors (White and Patton, 2023).

### 2.1 The zebrafish brain and cerebrovascular network as models

The zebrafish brain possesses different vascular features that confer to it a high degree of similarity to the mammalian brain. It is covered and protected by the cranial bones of the skull, and a recent study demonstrates the existence of a functional lymphatic system that runs in the ventral aspect of the skull, above the brain, similar to mammals (Castranova et al., 2020). Anatomically, the zebrafish brain is composed of the forebrain, midbrain and hindbrain regions. It contains conserved specialized regions such as the olfactory bulb, hypothalamus and cerebellum (Jurisch-Yaksi et al., 2020) as well as ventricles, choroid-plexus and cerebrospinal-fluid (CSF) (Korzh, 2023; Jeong et al., 2024). Its significantly smaller size compared to brains of other models, such as rodents, is an important advantage that facilitates high-resolution anatomical studies of entire intact brains (Kirst et al., 2020a; Ji et al., 2021a; Wu et al., 2022) and high-throughput molecular studies such as spatial transcriptomics. While the anatomy of the arterial anastomotic system that supplies blood to the zebrafish brain, namely, the Circle of Willis, seems to resemble that of mammals (Cheng et al., 2024), it is still not clear how the mature vascular network is organized inside the adult fish brain, and a detailed description of the vascular system of the zebrafish adult brain is still not available. For example, how superficial and penetrating arteries and veins are spatially organized, and whether the branching patterns that are hallmark of the mammalian cerebrovascular network are paralleled in zebrafish. Nevertheless, data from several studies suggest that this cerebrovascular system is still developing in juvenile stages and forms an elaborate network of blood vessels (BV) in the adult fish (Bower et al., 2017; Venero Galanternik et al., 2017; Chen et al., 2024a; van Lessen et al., 2017). At the vascular level, BV of the zebrafish brain display a striking similarity to mammalian ones. Genetically stable transgenic fish lines that express fluorescent tissue-specific reporters have confirmed the presence of various populations of perivascular cells, i.e., those that are found around the endothelium. Perivascular macrophages and mural cells, comprising Vascular Smooth Muscle Cells (VSMC) and pericytes, all have been visualized around BV in zebrafish brains (Cheng et al., 2024; Bower et al., 2017; Venero Galanternik et al., 2017; van Lessen et al., 2017; Ando et al., 2021). One of the most striking features of the mammalian brain is the presence of the BBB, that provides an isolated environment for parenchymal neural tissue. In this respect, zebrafish brains display barrier properties from three days-post-fertilization (dpf) onwards (Xie et al., 2010; Quiñonez-Silvero et al., 2020), further supported by the presence of hallmark cellular and molecular elements that comprise the BBB (O'Brown et al., 2018).

## 3 Zebrafish as an ageing model

**FIGURE 1:**
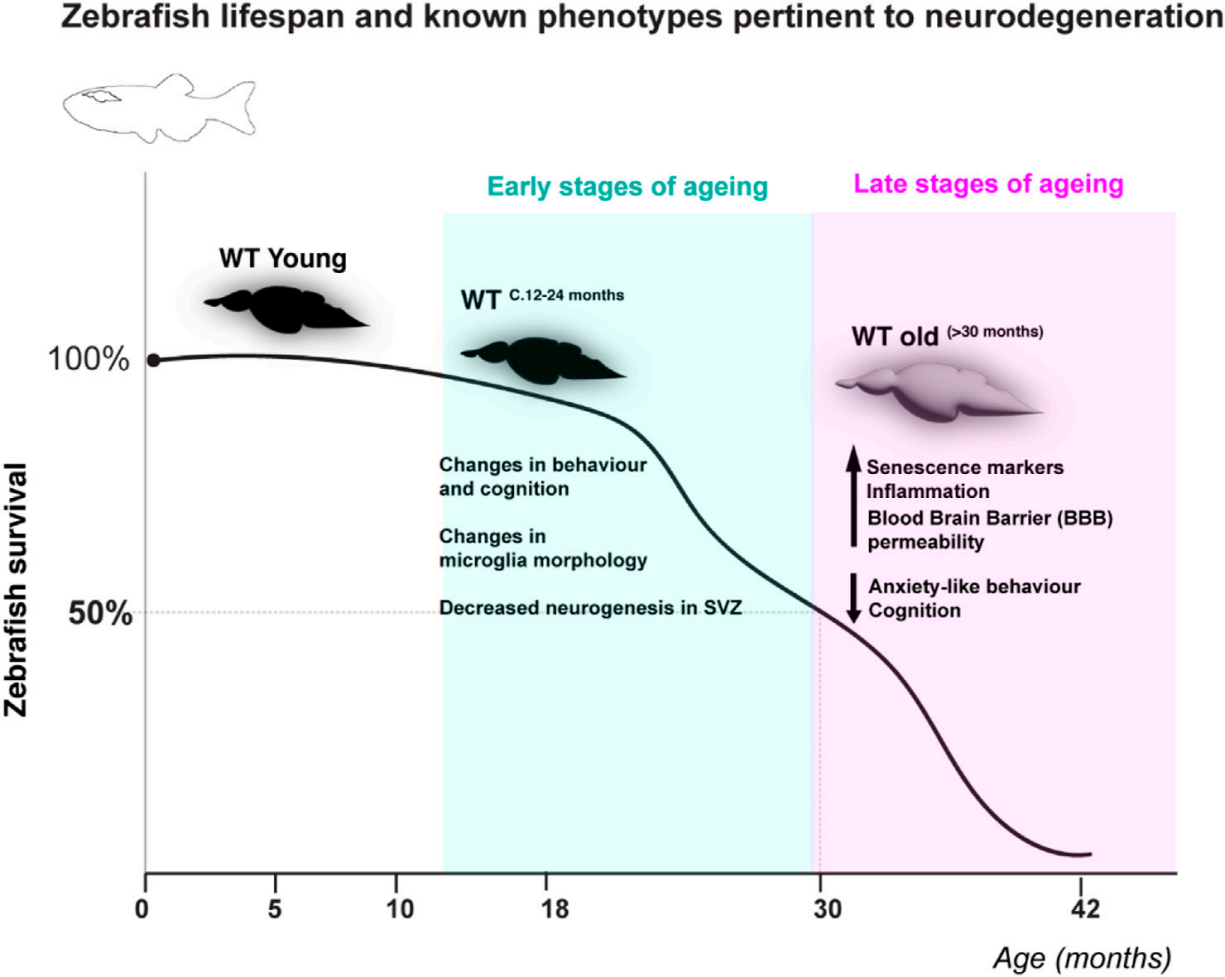
Zebrafish lifespan and known phenotypes pertinent to neurodegeneration. WT zebrafish have been shown to live up to at c.42 months in the lab and have a half-life of c. 30–31 months (cartoon depiction of lifespan based on (Carneiro et al., 2016a; Carneiro et al., 2016b). Despite many mechanisms and phenotypes pertinent to neurodegeneration remaining unknown, changes in behaviour, microglia morphology and neurogenesis have been described at early stages of zebrafish ageing (c.12–24 months), and ageing hallmarks such as senescence and inflammation, as well as increased blood brain barrier permeability have been suggested to occur at later stages of ageing (Espigares et al., 2021; Van Houcke et al., 2015; Raquel et al., 2024; Kishi et al., 2008; Hudock and Kenney, 2024; Kacprzak et al., 2017; Zambusi et al., 2020; Yu et al., 2006).

Zebrafish have emerged in the past c.10 years as a valuable complementary model to study human-relevant ageing mechanisms, such as telomerase-dependencies (Henriques et al., 2013; Carneiro et al., 2016a). The zebrafish value as an ageing model and fundamental guidelines on how to use both wild type and premature ageing models such as the telomerase (*tert* ^*−/−*^ *)* mutant have been extensively reviewed elsewhere (Henriques and Ferreira, 2024; Carneiro et al., 2016b; Pam et al., 2024; Kishi et al., 2003). Briefly, zebrafish display a time-dependent accumulation of age-associated phenotypes reminiscent of human ageing (Carneiro et al., 2016b; Pam et al., 2024; Kishi et al., 2003; Kishi et al., 2009). Zebrafish lifespan is 3 years on average and over 5 years in laboratory conditions (Kishi et al., 2009). Different tissues were shown to age at different rates, with highly proliferative tissues such as the testis and gut displaying age-associated dysfunction in WT fish from as early as 18 and 24 months, respectively. Specifically, zebrafish male fertility declines from 18 months onwards and the gut displays significantly increased DNA damage, senescence and inflammation by 24 months of age, which precedes increased gut leakiness in older ages (>30 months) (Ellis et al., 2022). Importantly, telomere shortening was shown to precede increased DNA damage response activation and a variety of phenotypes of old age (Carneiro et al., 2016a). Examples of these are: retina degeneration (Carneiro et al., 2016a), spine curvature, infections, loss of body mass and cancer (Carneiro et al., 2016a; Gerhard et al., 2002; Anchelin et al., 2013). Importantly, the cachectic/frail phenotype is underpinned by a significant decrease in subcutaneous adipose tissue and muscle fiber thickness (Carneiro et al., 2016a), as has been described in human frailty (Trueland, 2013; Takahashi et al., 2017). For simplicity, and to align with human phenotypes, we would normally consider zebrafish “old” at the age at which most of the fish present age-associated phenotypes, such as cachexia, loss of body mass and curvature of the spine. These phenotypes develop close to the time of death and are observed at >30 months of age in WT, which can be considered late stages of ageing (Figure 1), and at >12 months in *tert* ^*−/−31, 41*^. Telomerase deficiency in zebrafish is therefore reminiscent of the human scenario, where telomerase loss-of-function mutations or mutations affecting telomere stability lead to premature ageing syndromes (Alter et al., 2012; Hofer et al., 2005). Zebrafish also display altered behavior with ageing and show signs of neurodegeneration (Espigares et al., 2021; Van Houcke et al., 2015; Kishi et al., 2008; Hudock and Kenney, 2024; Kacprzak et al., 2017; Zambusi et al., 2020; Yu et al., 2006) (Figure 1), including telomerase-dependent neuroinflammation, senescence markers and increased blood brain barrier permeability with ageing (Raquel et al., 2024). How cerebrovasculature may change with ageing and the mechanisms involved remain, however, largely unexplored in zebrafish. Below we highlight some properties that we believe make zebrafish a valuable and exciting to study cerebrovascular ageing.

### 3.1 Using zebrafish in cerebrovascular ageing research

The zebrafish brain could be a valuable and useful model for studying cerebrovascular ageing for at least two main reasons. The first is that, as mentioned above, its cerebrovascular network displays important elementary similarities to the mammalian brain, at multiple levels. The second is related to the practical advantages that this model offers. Zebrafish start displaying age-associated phenotypes from c.18 months onwards, depending on the tissue, which can be considered an early stage of ageing (Figure 1). Recent advances in *in-vivo* optical microscopy of live adult fish (Castranova et al., 2022), including non-invasive imaging of the brain (Mizoguchi et al., 2023; Hamilton et al., 2022), now make it feasible to sample, at regular intervals, cerebral vessels of single fish as they age. This would enable to compare the structure of the vascular network over time, and to understand when and how it changes, for example, to detect changes in vascular density or vessel calibers throughout the lifecourse, all the way until old age. This can be further complemented by imaging of the blood flow in real time, adding a crucial functional aspect, thus linking structural and hemodynamic changes. Indeed, such a study recently reported on structural and functional changes in the ageing zebrafish telencephalon (Mizoguchi et al., 2023), in-line with results obtained in mice (Bennett et al., 2024). It will be interesting to examine whether reconstructing the zebrafish cerebrovasculature, using high-resolution three-dimensional imaging methods, will reveal heterogeneities in vascular densities across different brain regions as were found in the mouse brain (Kirst et al., 2020b; Ji et al., 2021b; Wu et al., 2022). This is likely to be easier to achieve in zebrafish due to their small size. Moreover, the wealth of available vascular-specific fluorescent transgenic lines and mutants (Ando et al., 2021; Choe et al., 2021) offers the possibility to examine what happens also to the different vascular components, and especially the BBB, throughout life and into ageing. Other advantages of zebrafish that could be used in the context of ageing are generation of novel transgenic reporter or mutant lines that target specific age-related genes. In zebrafish this is now performed more efficiently with Crispr/Cas9, and can be corroborated with high-throughput screens for potential drugs or molecules. Finally, a unique property of zebrafish is that even at adult stages, damaged tissue or organs can regenerate following injury (Marques et al., 2019). This is also the case for the zebrafish brain, where damaged tissue regenerates with minimal or absent scarring (Chen et al., 2024b). Little is currently known on how the vascular system regenerates in the brain, and this property of zebrafish can be used to examine if and how the regenerative efficacy is affected by age. Importantly, regeneration studies so far have relied on several damage paradigms, including phototoxic (Ranski et al., 2018) and ouabain-induced lesions (Mitchell et al., 2019), which induce rapid cell death post-insult. While high-intensity acute damage models are suitable for exploring the cellular and molecular mechanisms underpinning tissue regenerative potential, they do not test whether this regenerative response is also occurring with natural ageing, and so the use of a suitable ageing model is essential. In fact, previous work highlights how natural ageing does not elicit the same regenerative response as high-intensity acute damage in the zebrafish retina (Martins et al., 2022).

**FIGURE 2:**
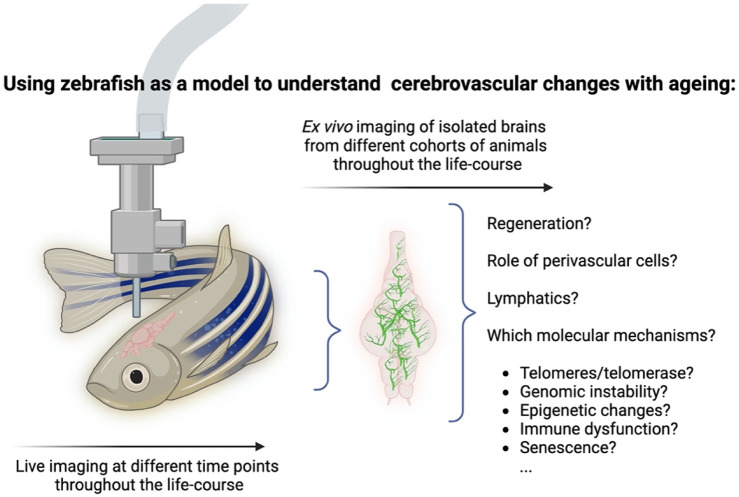
Using zebrafish as a model to understand cerebrovascular changes with ageing. The zebrafish offers unique characteristics that make it amenable to study cerebrovascular ageing throughout life. Either using live imaging techniques or via *ex-vivo* imaging of isolated brains from cohorts of animals throughout the life-course. Combinations of cell-specific fluorescent reporter lines combined with premature models of ageing should allow the identification of key mechanisms underpinning degeneration. Created in BioRender. Henriques, C. (2025) https://BioRender.com/w52z573. Image not to scale and cerebrovascular network depiction is not anatomically representative- for illustration purposes only.

Zebrafish is therefore a unique model in that it allows the identification of age-associated mechanisms underpinning degeneration and regeneration in the same tissue, using different techniques (Figure 2).

## 4 Conclusion

A number of studies in recent years highlight the inter- and intra-heterogenic nature of ageing, i.e., the differences between individuals and within the same individual. This would suggest that there are elements such as genetic variations, environmental factors and inherent physiological determinants that come into play in this lengthy process, and that a variety of interactions must be considered in order to describe ageing and its underlying mechanisms. This may seem surprising given that hallmark molecular and cellular signatures to ageing have been identified, which were suggested to underpin age-associated degeneration and disease (López-Otín et al., 2023). Nevertheless, it is recognised that an important hurdle in ageing research is the ability to link what is known to occur at molecular and cellular levels to the tissue, organ and entire organism levels. In the specific context of vascular ageing, it will be important to understand some of the following questions, such as if and how molecular and genomic-level events (instabilities, epigenetic and telomeric changes) and cellular-level events (mitochondrial dysfunction, senescence, exhaustion of stem cells) lead over time to changes in the structure and function of the vascular system; what are the pathways that are involved; and do all vascular beds and tissues age in the same manner, across different organs. In order to tackle such questions, it is necessary to use an appropriate model. What makes any model a suitable one for studying ageing in general, and cerebrovascular ageing in particular? The most basic requisite is that it has to display signatures of ageing, where a gradual deterioration in the function and structure of tissues and organs is the most obvious phenotypical change. Ideally, it should also display a large set of common features with other models, which would enable to draw conclusions across species. Finally, pertaining to inherent features of the model itself, it should enable to study ageing over time, at a relevant and meaningful temporal resolution, which would enable to construct a chronological “atlas” of the process, in the same animal. In this respect, the zebrafish cerebrovascular network may be well suited to examine the changes that are associated with ageing throughout the lifecourse, over time, and to link molecular and cellular changes to an overall structural and functional vascular decline (Figure 2). Using the advantages of this model to complement existing models could open new research paths and greatly enhance our understanding, at a higher temporal resolution, of how the vascular network evolves over time, what are the molecular and cellular that underlie these events, and if and how these series of events can be modified or manipulated to induce a beneficial outcome.

## Data Availability

The original contributions presented in the study are included in the article/supplementary material, further inquiries can be directed to the corresponding authors.
